# Frequency of All Types of Colorectal Tumors in the Patients Referred to Selected Hospitals in Tehran

**DOI:** 10.5812/ircmj.4026

**Published:** 2013-06-05

**Authors:** Farzaneh Golfam, Parisa Golfam, Zeinab Neghabi

**Affiliations:** 1 Mostafa Khomeini Hospital, Department of Surgery, Shahed University, Tehran, IR Iran; 2 Imam Reza Hospitah, Kermanshah University of Medical Sciences, Kermanshah, IR Iran; 3 Student Research Committee, Shahed University, Tehran, IR Iran

**Keywords:** Colorectal Cancer, Adenocarcinoma, Colon

## Abstract

**Background:**

Colorectal cancer (CRC) is one of the most common cancers worldwide which is not extensively researched in Iran.

**Objectives:**

The present study aims to investigate the epidemiologic characteristics of CRC in patients referred to selected hospitals of Tehran University of Medical Sciences.

**Patients and Methods:**

In this descriptive-analytic study 218 patients with colorectal cancer were investigated. Data were collected via reviewing recorded pathologic results of patients of these hospitals which then were analyzed by univariant methods.

**Results:**

Among 218 patients, 140 (64.2%) were male and 78 (35.8%) were female. Of all patients, 132 (60.0%) suffered from tumors in rectum; 38 (17.4%) in sigmoid; 12 (5.5%) in descending colon; 10 (4.6%) in transverse colon and 26 (11.9%) in ascending colon and cecum. Tumors were well differentiated in 134 patients (61.5%), moderately in 62 cases (28.4%) and poorly differentiated in 22 patients (10.1%). There was no significant difference between males and females regarding the location and degree of tumor differentiation.

**Conclusions:**

Regarding high prevalence of colorectal cancer in Iran and potential environmental and genetic factors, surveillance must be considered for this disease. Its risk factors such as diet, life style and low physical activity should be evaluated and screening should start at younger ages.

## 1. Background

Nowadays, cancer as an important problem has influenced public health and governments’ sanitary measures (1). Colorectal cancer (CRC) is one of the most important cancers and the second cause of cancer-related mortality in the United States (2, 3). Annually, about one million new cases of colorectal cancer are diagnosed all over the world with a mortality rate amounting to approximately 500,000 deaths. Recent reports show that colorectal cancer has been introduced as the most common cancer in people over 75 years in the United States (3). Epidemiologic characteristics of colorectal cancers are different in various parts of the world. For instance, incidence rate of this cancer is annually reported to be about 30-50 cases in a 100,000 population in the north America and Europe whereas incidence rate is estimated 3-7 cases in a 100,000 population in the Middle Eastern communities (4, 5). In European countries, 2-8% of all colorectal cancers occur in people below 40 years (4, 6-9) whereas in the Middle Eastern countries 15-35% of patients with colorectal cancer are below age 40 (10-12). According to the annual reports of the Cancer Institute, an offshoot of Diseases Management Center, colorectal cancer is the third common cancer in Iranian women and the fifth common cancer in Iranian men (13). Incidence rate of the disease has increased during the past 25 years in Iran (14), due seemingly to a change in life style in urban areas and adopting a Western life style. Despite the significance of this rapid rise information concerning epidemiologic features of CRC in Iran is limited and more research as the present study is still required to investigate epidemiologic characteristics of CRC in Iran. 

## 2. Objectives

The main objective of present study is to explore epidemiologic characteristics of CRC in the patients referred to selected hospitals of Tehran University of Medical Sciences in the period between 2001 and 2010.

## 3. Patients and Methods

As a cross sectional research, the present study has been conducted in Imam Khomeini and Shariati Hospitals in Tehran within 2009-2010. The population included patients with colorectal cancers with a documented pathology report in which information about location, type, and differentiation of tumor have been recorded. Patients without such information were excluded from the study. We used easy nonrandomized method for sampling in which all patients who referred to Imam Khomeini and Shariati Hospitals within 2009-2010 were enrolled. According to first type error equal 5%, accuracy equal 0.06 and well differentiation rate of 40% for tumor in previous studies we calculated sample size as 220 patients. Data were recorded in data collecting forms, a sample of which is available in the appendix. This form contained information about age, gender, pathologic findings and tumor location. We evaluated the information concerning all patients who were introduced to the pathology ward and demonstrated histopathology of their lesions. All acquired data were recorded in collecting forms. After coding, data were analyzed by SPSS 15 statistical software. For statistical analysis, we used frequency and rates for qualitative variables and mean and standard deviation for quantitative variables. Chi-2 was used for comparing rates while independent t-test and ANOVA were used for comparing means. P-values less than 0.05 were considered as significance level. 

## 4. Results

At the beginning of the study, 244 patients were enrolled. Twenty six cases were excluded because their mass was non-specific or due to incomplete data about their disease. The remaining 218 patients consisting of 140 males (64.2%) and 78 females (35.8%) were evaluated. The Mean age was 53.3 ± 8.8 (mean ± SD) ranging between 15 and 87 years. Among 218 investigated patients 30 (13.8%) were 40 or less than 40 years old. Frequency of tumors based on tumor location has been summarized in Table 1. 

**Table 1.** Frequency of Tumors Based on Tumor Location

Location of Tumor	Frequency	Rates (%)	Accumulative, %
**Rectum**	132	60.6	60.6
**Sigmoid**	38	17.4	78
**Descending colon**	12	5.5	83.5
**Transverse colon**	10	4.6	88.1
**Ascending colon and cecum**	26	11.9	100
**Total**	218	100	

According to the current study, 196 (89.9%) cases were adenocarcinoma. The 22 remainder included SCC in 4 (1.8%) cases, lymphoma in 4 (1.8%) cases, and neuroendocrine carcinoma in 2 (0.9%) cases. In addition, 12 cases (5.6%) were undifferentiated tumors. Concerning the degree of differentiation, tumors were well differentiated in 134 patients (61.5%), moderately differentiated in 62 cases (28.4%) and poorly differentiated in 22 patients (10.1%). To evaluate the association between age and degree of tumor differentiation, we compared mean age of patients based on tumor differentiation rates. Comparison showed that the mean age in patients with well differentiated tumor was 54.8 ± 7.5, in patients with moderately differentiated tumor was 52.2 ± 6.8 and in patients with poorly differentiated tumor was 48.5 ± 7.2 years. Comparison of these results showed no statistically significant difference (P = 0.563). Additionally, the mean age was 56.6 ± 9.1 in patients with right side cancers (cecum and ascending colon), 58.1 ± 5.8 in patients with transverse colon tumors , 52.1 ± 4.7 in patients with left side tumors ( descending colon and sigmoid) and 50.3 ± 6.4 years old in patients with rectal tumor . Comparison of these means showed no statistically significant difference (P = 0.753). Degree of tumor differentiation based on gender of patients is indicated in Table 2 below. As it is observed no statistically significant difference (P = 0.753) is indicated. 

**Table 2.** Degree of Tumor Differentiation Based on Gender of Patients

Degree Of Tumor Differentiation	Male, No. (%)	Female, No. (%)	Total, No. (%)
**Well differentiated**	82 (61.2)	52 (38.8)	124 (100)
**Moderately differentiated**	44 (64.5)	18 (35.5)	62 (100)
**Poorly differentiated**	8 (36.4)	14 (63.6)	22 (100)
**Total**	78 (35.8)	140 (64.2)	218 (100)

Rates and frequency of tumor location based on gender are summarized in Table 3, where no statistically significant difference (0.268) is indicated. 

**Table 3.** Rates and Frequency of Tumor Location Based on Patient's Gender

Tumor location	Male, No. (%)	Female, No. (%)	Total, No. (%)
**Rectum**	90 (68.2)	42 (32.8)	132 (100)
**Sigmoid**	22 (52.6)	16 (47.4)	38 (100)
**Descending colon**	6 (50)	6 (50)	12 (100)
**Transverse colon**	80 (80)	2 (20)	10 (100)
**Ascending tumor and cecum**	14 (53.8)	12 (46.2)	26 (100)
**Total**	140 (64.2)	78 (35.8)	218 (100)

## 5. Discussion

Results of our study indicated that the rate of male patients has been more than female patients (male to female rate = 1.8:1). Similarly, in the majority of previous studies about gender of CRC the male predominance has been reported. In Salari et al. study (Yazd Shahid Sadooghi University of Medical Sciences and Treatment-Health Services) 55.5% of patients were male whereas 44.5% were female (15). Also , In Jalali and colleagues’ investigation at Tehran University of Medical Sciences 58% of CRC cases were male and 42% were female (16). Similarly in a study performed at Arak University of Medical Sciences by Fateh and Amini 55% of CRC patients were male and 45% were female (17). Molanaie and colleagues in Kordestan University of Medical Sciences showed that 61% of cases were male and 39% were female (18). The mean age of patients in our study was 53 years old with a range of 15- 87 years. According to the standard deviation, the majority of our patients were 45 to 65 years old. As far as age is concerned, the results of the present study are somewhat similar to results of previous studies. For instance, Salari et al. reported a range of 28-94 years for age of CRC patients. In Salari's study 127 patients were 60-69 years old whereas 7.5% of them were younger than 40 years old (15). Also, In Jalali and colleagues’ study, (16) the mean age was 51 ± 15 years (mean ± standard deviation). Similarly, in Fateh and coworkers’ investigation only 17.5% of patients were younger than 40 years old (17). In addition, Fakheri et al. reported that mean age of CRC patients was 53 years with a standard deviation equal to 15 years (19). Furthermore, results of the present study indicated that the tumor type is adenocarcinoma in most of our patients studied and lower rates of patients had other types of tumor. In addition, the most common sites for tumor were rectum and thereafter sigmoid. As far as tumor site is concerned, there was no difference between men and women. In addition, there was no significant association between age of patients and the pathologic pattern mentioned. On the other hand, both young and old patients had somewhat followed the same pathologic pattern. In Dr Salari´s study, there were 186 (97.4%) cases with adenocarcinoma and 5 (2.6%) cases with lymphoma (15). In Fateh and colleagues’ study, adenocarcinoma was the most common tumor and rectum was the most common site of tumor (17). Likewise, in Safai et al. investigation, adenocarcinoma was the most common type of tumor and in more than 39% cases was well differentiated; also, there was no significant difference between men and women. In Dr Molanae’s study regarding tumor type, 90% were adenocarcinoma, 7% were lymphoma and 3% were carcinoid tumors (18). All findings of the present study demonstrated that CRC pattern has not notably changed during recent 20 years. In addition, CRC pattern in our patients is similar to the other parts of Iran which is seemingly due to the fact that hospitals in Tehran University of Medical Sciences are mostly referral hospitals with patients from all over the country. It should be noted here that alteration in people lifestyle especially diet may have an effect on colorectal cancer features. As a result it is able to change CRC patterns in the future. 
